# Mitochondria and Lysosomes Participate in Vip3Aa-Induced *Spodoptera frugiperda* Sf9 Cell Apoptosis

**DOI:** 10.3390/toxins12020116

**Published:** 2020-02-13

**Authors:** Xiaoyue Hou, Lu Han, Baoju An, Yanli Zhang, Zhanglei Cao, Yunda Zhan, Xia Cai, Bing Yan, Jun Cai

**Affiliations:** 1 Department of Microbiology, College of Life Sciences, Nankai University, Tianjin 300071, China; xiaoyuehou@mail.nankai.edu.cn (X.H.); luhan0325@mail.nankai.edu.cn (L.H.); 1120190491@mail.nankai.edu.cn (B.A.); Yanlizhang@mail.nankai.edu.cn (Y.Z.); caozhanglei@mail.nankai.edu.cn (Z.C.); yundazhan@mail.nankai.edu.cn (Y.Z.); xiacai@mail.nankai.edu.cn (X.C.); iceyan@nankai.edu.cn (B.Y.); 2 Key Laboratory of Molecular Microbiology and Technology, Ministry of Education, Tianjin 300071, China; 3 Tianjin Key Laboratory of Microbial Functional Genomics, Tianjin 300071, China

**Keywords:** Vip3Aa, lysosome, mitochondria, apoptosis, Sf9 cells

## Abstract

Vip3Aa, a soluble protein produced by certain *Bacillus thuringiensis* strains, is capable of inducing apoptosis in Sf9 cells. However, the apoptosis mechanism triggered by Vip3Aa is unclear. In this study, we found that Vip3Aa induces mitochondrial dysfunction, as evidenced by signs of collapse of mitochondrial membrane potential, accumulation of reactive oxygen species, release of cytochrome c, and caspase-9 and -3 activation. Meanwhile, our results indicated that Vip3Aa reduces the ability of lysosomes in Sf9 cells to retain acridine orange. Moreover, pretreatment with Z-Phe-Tyr-CHO (a cathepsin L inhibitor) or pepstatin (a cathepsin D inhibitor) increased Sf9 cell viability, reduced cytochrome c release, and decreased caspase-9 and -3 activity. In conclusion, our findings suggested that Vip3Aa promotes Sf9 cell apoptosis by mitochondrial dysfunction, and lysosomes also play a vital role in the action of Vip3Aa.

## 1. Introduction

Vip3Aa is a protein produced by *Bacillus thuringiensis* (*Bt*) during vegetative growth. It can bind to the brush border membrane vesicles (BBMV) specifically in susceptible and non-susceptible insects [1,2,3]. Moreover, the brush border membrane binding sites of Vip3Aa are different from those of insecticidal crystal proteins (ICPs), and Vip3Aa could extend its activity to pests non-susceptible to ICPs. Consequently, it is widely accepted that Vip3Aa can not only broaden the insecticidal spectrum, it may also delay the resistance development in insects [3,4,5]. Thus, Vip3Aa is considered a second-generation insecticidal toxin and has been used in genetically modified crops, such as *Bt* cotton and *Bt* corn products [6].

The pore-forming model is generally accepted to explain the virulence of ICPs [7] and Lee et al. [3] corroborated that the Vip3 proteins share a similar mode of action. In short, the Vip3 proteins (protoxins) are ingested by the insect and activated to the active form (act-Vip3A) by the midgut proteases. After that, the act-Vip3A binds to its receptor on the BBMV and exerts toxicity to the midgut cells, eventually leading to the death of the pests. Additionally, Kunthic et al. [8] found that the pH could regulate the properties of the tetramer made by the act-Vip3Aa, which further supported the pore-forming model and suggested that the pH could regulate the post-binding events such as membrane insertion or pore formation. Regarding the binding sites of the Vip3Aa, recent research has found some proteins interacting with Vip3Aa that are closely related to cell toxicity in *Spodoptera frugiperda* cells, such as S2, SR-C, and FGFR [5,9,10]. Additionally, Jiang et al. [9] found that the toxicity of Vip3Aa to Sf9 cells correlated with its endocytosis mediated by Sf-SR-C and that internalization is essential for Vip3Aa to exert its toxic effects. 

Bel et al. [11] showed Vip3Aa provoked a wide transcriptional response in *Spodoptera exigua* larvae. The upregulated genes were involved in innate immune response and pathogen response, while the downregulated ones were mainly related to metabolism. However, genes related to the action of ICPs were found to be slightly overexpressed. Crava et al. [12] further indicated that Vip3Aa upregulated genes coding for antimicrobial peptides and lysozymes in *S. exigua* midgut. Ayra-Pardo et al. [13] reported a transcriptomic study, showing that the decreased translation rate could be an important adaptation for Vip3Aa resistance in *Heliothis virescens*. Hernández-Martinez et al. [14] suggested Vip3Aa could activate different insect response pathways that trigger the regulation of some genes, APN shedding, and apoptotic cell death. These results suggest that there are other mechanisms that are participating in cell death apart from the pore-forming model. Jiang et al. [15] observed that the Vip3Aa-treated Sf9 cells had some apoptosis characteristics, such as DNA breakage, mitochondrial membrane potential (*ΔΨm*) collapse, and Sf-caspase-1 activation. Hernández-Martinez et al. [14] confirmed that there was apoptosis occurrence in midgut epithelial cells when *S. exigua* larvae were treated with Vip3Aa and Vip3Ca. However, how Vip3Aa induces apoptosis is unclear and further experiments will be needed to determine the underlying mechanism.

Apoptosis is indispensable to the homeostasis and development of organisms [16]. Bcl-2 family proteins are crucial regulators of cell survival and cell death. They are divided into anti- and pro-apoptotic proteins. After apoptotic stimulation, Bax, a pro-apoptosis protein, can transfer to mitochondria, resulting in mitochondrial membrane permeability increase and cytochrome c release. The mitochondrion, a highly sensitive organelle, plays a critical role in apoptosis. Increased mitochondrial membrane permeabilization may represent the point of no return of the lethal stressors-induced signal [17]. Cytochrome c normally localizes in the inner mitochondrial membrane through weak electrostatic interactions with acidic phospholipids. When mitochondria permeability increases, it releases to the cytoplasm and subsequently activates the apoptotic cascades. Anti-apoptotic proteins, such as Bcl-2 and Bcl-XL, inhibit apoptosis by locally preventing *ΔΨm* loss [17,18]. Environmental stimuli may contribute to mitochondrial injury, which causes *ΔΨm* collapse, oxidative stress, resulting in increased cellular ROS, changed Bcl-2 family protein levels, and apoptosis factor release [19,20,21].

In this paper, we try to further explore the mechanism of Vip3Aa-induced apoptosis and probe the signaling pathways and molecules involved in Vip3Aa-induced cell death.

## 2. Results

### 2.1. The Effects of Vip3Aa on Sf9 Cell Viability and the Subcellular Localization of Vip3Aa in Sf9 Cells

**Figure 1:**
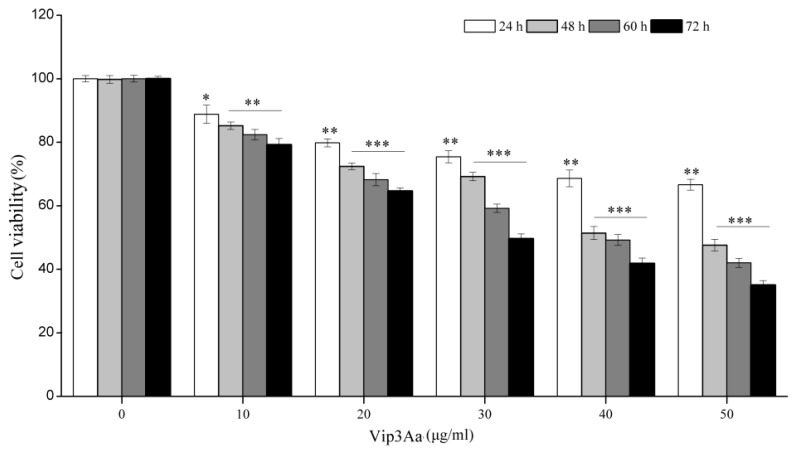
Viability impacts of Vip3Aa on Sf9 cells. The Sf9 cells were exposed to different concentrations of Vip3Aa for 24, 48, 60, and 72 h, respectively. Significant tests from the corresponding controls (without Vip3Aa treatment) are indexed via * *p* < 0.05, ** *p* < 0.01, and *** *p* < 0.001.

Sf9 cells were exposed to Vip3Aa (10, 20, 30, 40, or 50 μg/mL) for different times (24, 48, 60, and 72 h). Cell viability of Sf9 cells was assessed by the CCK-8 assay, by measuring the amount of orange–yellow formazan that is directly proportional to the number of living cells. As illustrated in Figure 1, when Sf9 cells were exposed to the same Vip3Aa concentration, the cell viability decreased as the time of treatment prolonged. If the Vip3Aa-treated time was the same, cell viability decreased with the increase of Vip3Aa concentration. Vip3Aa (final concentration, 40 μg/mL) treatment for 48 h reduced the cell viability of Sf9 cells to about 50%. Thus, the final concentration of Vip3Aa used in the following experiments was 40 μg/mL.

**Figure 2:**
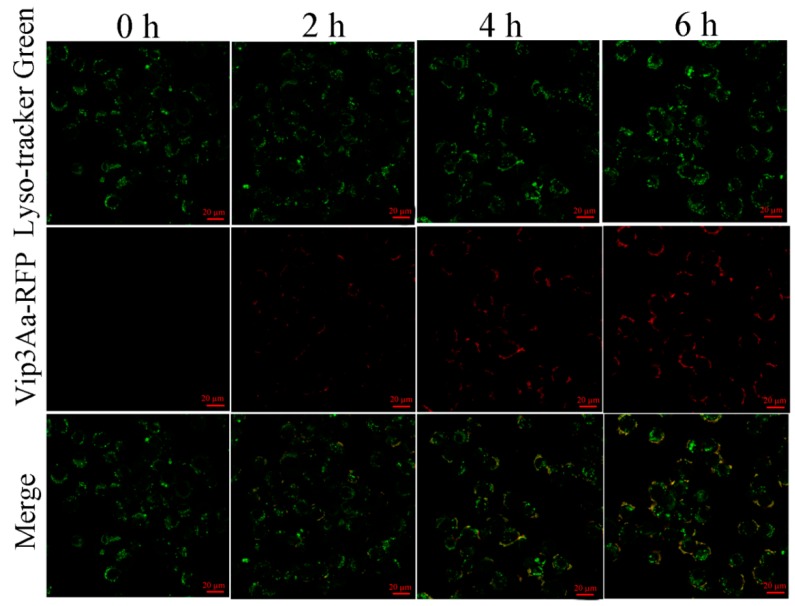
Co-localization of Vip3Aa and lysosomes in Sf9 cells. Cells were treated with Vip3Aa-RFP for 0, 2, 4, and 6 h, respectively, and were stained with fluorescent probe LysoSensor™ Green DND-189 at 28 ℃ for 45 min. Then, the cells were observed under a confocal laser scanning microscope. Scale bar, 20 µm.

Jiang et al. [9] revealed that Vip3Aa could enter into the Sf9 cells via endocytosis. Since the lysosome is the endpoint of endocytosis, we further explored the subcellular localization of Vip3Aa in Sf9 cells. Sf9 cells were exposed to Vip3Aa-RFP for different times (2, 4, and 6 h). As shown in Figure 2, there was abundant co-localization of Vip3Aa and lysosomes from 4 h after Vip3Aa treatment. These results suggested that lysosomes might be involved in the action of Vip3Aa.

### 2.2. Vip3Aa Impairs Mitochondrial Function and Induces Cytochrome c Release

**Figure 3:**
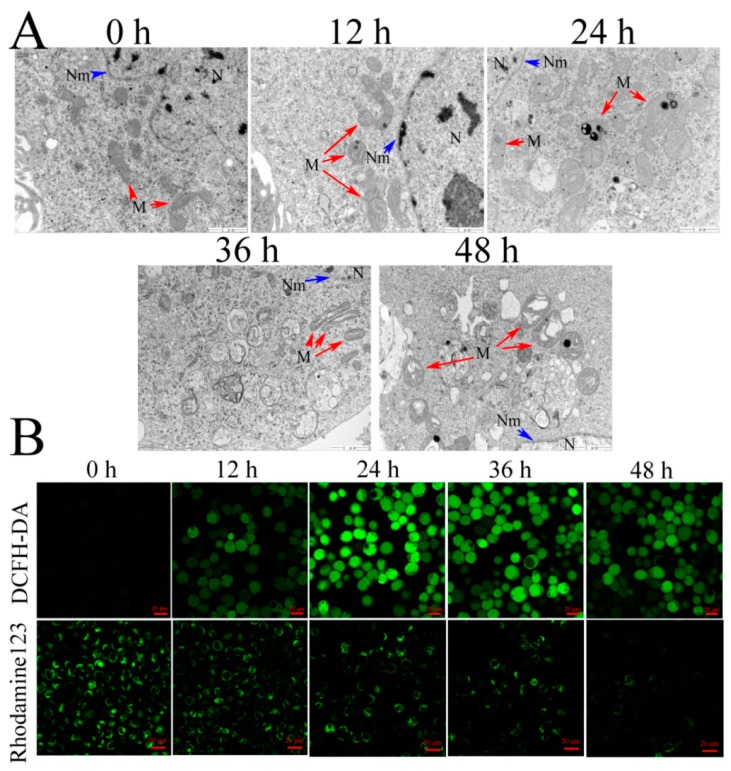
Effects of Vip3Aa on mitochondria in Sf9 cells. (**A**) Representative photographs of mitochondria ultrastructure in Sf9 cells after exposure to Vip3Aa, obtained by TEM. N, nucleus. Nm, nuclear membrane (blue arrows). M, mitochondria (red arrows). Magnification, 30000 ×. Scale bar, 1 μm. (**B**) Effects of Vip3Aa on ROS production and mitochondrial membrane potential (*ΔΨm*) in Sf9 cells, which were determined by the fluorescent probe DCFH-DA and Rhodamine123, respectively. Scale bar, 20 μm.

Mitochondria are the meeting point of many apoptotic signals, so we examined the mitochondrial ultrastructure (red arrows) in Sf9 cells by transmission electron microscope (TEM) (Figure 3A). The Vip3Aa-untreated cells showed a normal ultrastructure with an intact cristae structure. However, the number of twisted and swollen mitochondria increased from 12 to 24 h after Vip3Aa treatment. After 36 h, the outer membranes of most mitochondria were intact, but the cristae structures were disrupted. 

Mitochondria are not only the main source of endogenous ROS but also the “absorption bank” of ROS. Mitochondria play an essential role in regulating ROS metabolism. In turn, ROS also impacts the function of mitochondria [22]. We then explored the impact of Vip3Aa on the ROS in Sf9 cells. For Vip3Aa-treated cells, ROS levels increased within 12 h, peaked at 24 h, and then decreased (Figure 3B). 

Mitochondrial membrane potential (*ΔΨm*) plays a key role in mitochondria function [23]. Rhodamine 123 was used to detect *ΔΨm*. Results showed a significant decrease in *ΔΨm* appeared firstly at 24 h after Vip3Aa treatment, and the fluorescence intensity reduced to a lower level at 48 h (Figure 3B).

**Figure 4:**
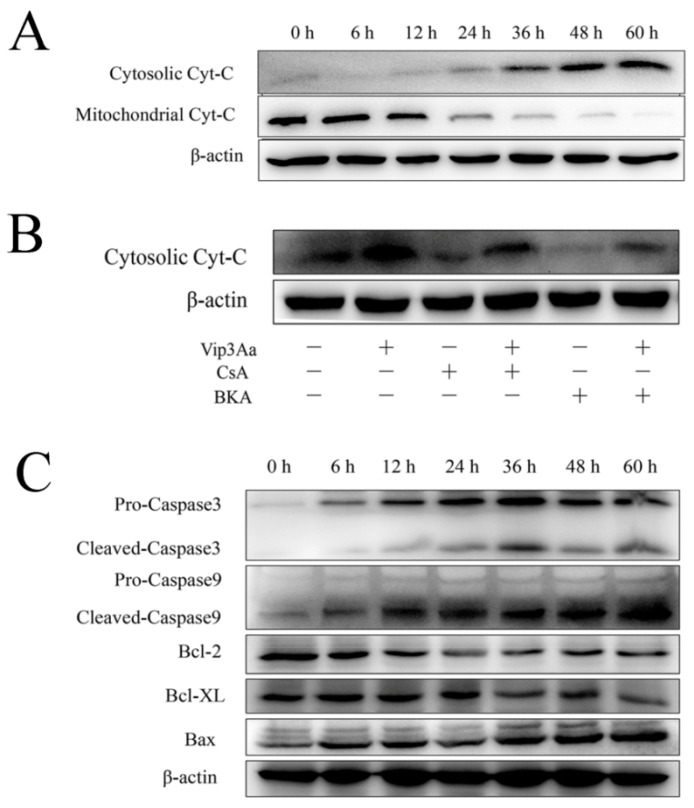
Subcellular distribution of cytochrome c and the levels of mitochondria-associated proteins in Sf9 cells after Vip3Aa treatment. (**A**) Subcellular distribution of cytochrome c after Vip3Aa treatment. Cytochrome c distribution in cytoplasm and mitochondria was detected by Western blotting. (**B**) Cytochrome c distribution in Sf9 cells after different treatments. CsA (5 μM) or BKA (10 μM) pretreated the Sf9 cells for 2 h before Vip3Aa treatment. (**C**) The mitochondria-associated proteins levels in Sf9 cells after Vip3Aa treatment. The original pictures of Western blotting (with protein maker) are shown in Supplementary Materials (Figure S1).

To determine the influence of Vip3Aa on cytochrome c distribution, we evaluated the cytochrome c content in the cytosol and mitochondria via Western blotting. As indicated in Figure 4A and Figure S1D, the cytochrome c content in the cytoplasm increased, while that in the mitochondria decreased significantly. Subsequently, this phenomenon became more apparent. 

To further explore the way of cytochrome c release, mitochondria permeability transition pore (mPTP) inhibitors, CsA and BKA, were used. Results showed that both inhibitors prevented cytochrome c release partly, while BKA exerted a stronger inhibitory effect than CsA did (Figure 4B). These results suggested that an mPTP-dependent mechanism was involved in cytochrome c release in Vip3Aa-treated Sf9 cells.

### 2.3. Effects of Vip3Aa on the Mitochondria-Associated Proteins Levels

The protein levels of the Bcl-2 family are related to mitochondrial function. As shown in Figure 4C and Figure S1E, Bax expression increased, while Bcl-2 and Bcl-XL decreased with the extension of Vip3Aa treatment time. 

In the mitochondrial pathway, cytochrome c release induces the formation of apoptotic protein complexes, which convert pro-caspase-9 into active caspase 9. Subsequently, caspase-9 will further activate caspase-3 and leads to cell apoptosis. So, we investigated whether caspase activation was involved in the Vip3Aa-induced cell death. Results suggested that the levels of cleaved-caspase-9 and cleaved-caspase-3 increased in different degrees with the extension of Vip3Aa treatment time (Figure 4C and Figure S1F). Additionally, when cytochrome c in the cytosol increased significantly, the protein level of cleaved-caspase increased accordingly. These results indicated that Vip3Aa induced dysregulation of mitochondria-associated proteins and subsequently led to the activation of caspases.

### 2.4. Effects of Vip3Aa on Lysosome Morphological and Physicochemical Property 

**Figure 5:**
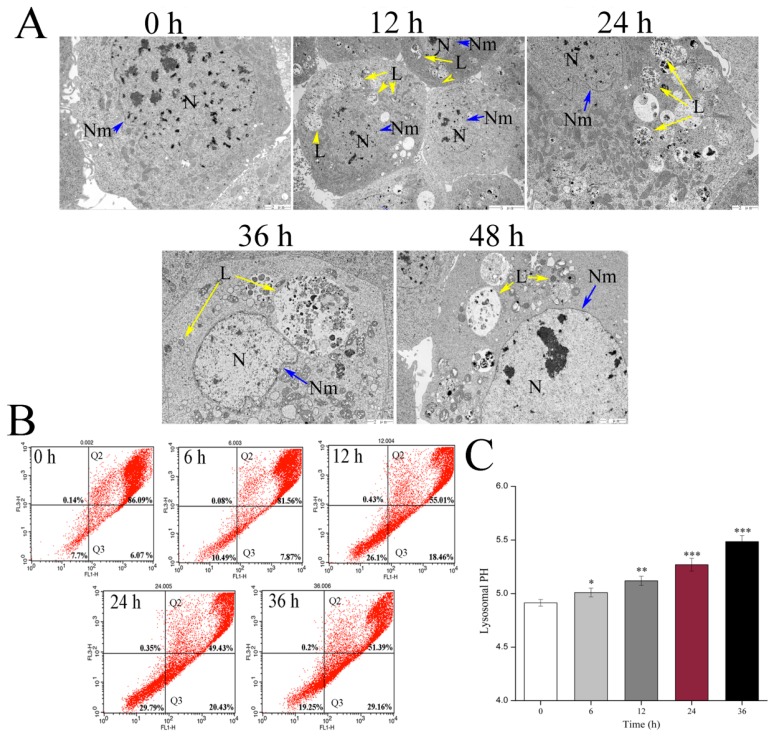
Effects of Vip3Aa on lysosomes in Sf9 cells. (**A**) Representative photographs of lysosomes ultrastructure in Sf9 cells after exposure to Vip3Aa, obtained by TEM. N, nucleus. Nm, nuclear membrane (blue arrows). L, lysosomes (yellow arrows). Magnification, 0 h, 24 h, 36 h, and 48 h, 10000 ×; 12 h, 5000 ×. (**B**) The physicochemical property of lysosomes was detected by acridine orange (AO) staining in Sf9 cells. (**C**) The lysosomal pH in Sf9 cells was detected using LysoSensor Yellow/Blue DND-160. Significant tests from the corresponding controls (without Vip3Aa treatment) are indicated by * *p* < 0.05, ** *p* < 0.01, and *** *p* < 0.001.

Several lines of evidence suggest that the lysosomal pathway contributes to apoptosis. To explore the impact of Vip3Aa on lysosomes, we observed the lysosomal ultrastructure in Sf9 cells by TEM (Figure 5A). The control cells showed a few lysosomes and the cytoplasm was homogeneous. However, the type of lysosomes in the Vip3Aa-treated cells became diverse, and some lysosomes increased distinctly in volume. Meanwhile, we measured the ability of lysosomes to retain acridine orange (AO). As shown in Figure 5B, the fluorescence signals for Sf9 cells were mostly kept in the Q2 region (normal cells), while the percentage of the Q3 region (cells with weak lysosomes) increased from 6.07% to 29.16% with the prolongation of Vip3Aa treatment time. These data showed that Vip3Aa increased the proportion of cells with abnormal lysosomes, and the ability of these lysosomes to keep AO was poor.

Additionally, we also measured lysosomal pH to further study the impact of Vip3Aa on lysosomes. The lysosome pH value of the Vip3Aa-untreated cells was estimated at 4.91, whereas the lysosomal pH increased at 5.48 after Sf9 cells were exposed to Vip3Aa for 36 h (Figure 5C).

### 2.5. The Relationship between Sf9 Cell Cathepsins and Vip3Aa-Induced Apoptosis and Cytotoxicity

Lysosomes could be involved in apoptosis via lysosomal proteases, especially cathepsins. Therefore, we measured the mRNA level of cathepsins B, L, and D, which are the significant proteins in the lysosome function. The results indicated that the expression levels of cathepsins (L and D) increased differently depending on the cathepsins analyzed (Figure S2). The mRNA level of cathepsin L and cathepsin D peaked at 36 and 6 h, respectively. However, the expression level of cathepsin B had little change after the cells exposed to Vip3Aa in all the times analyzed. 

**Figure 6:**
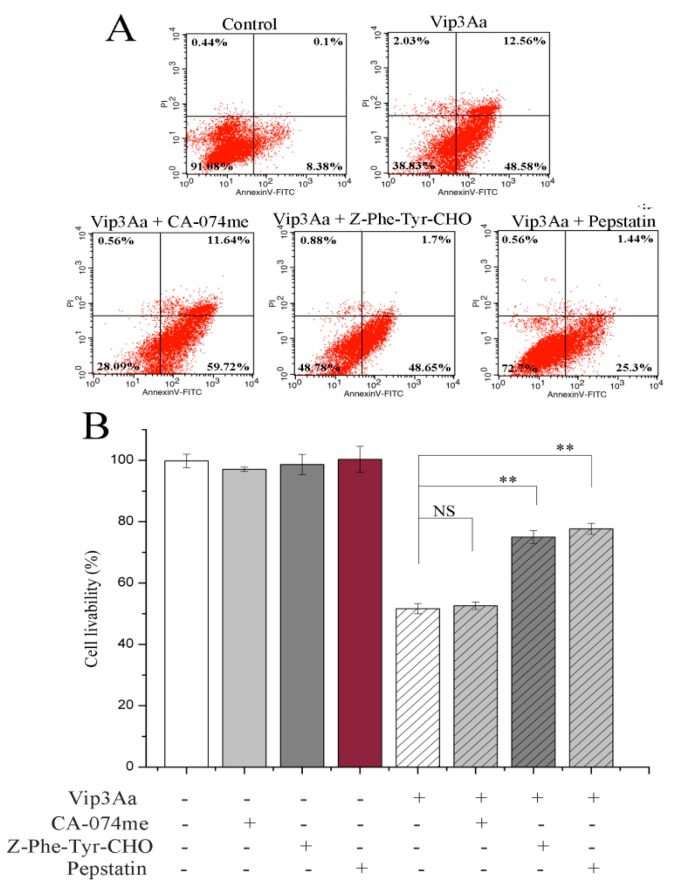
Effects of cathepsin inhibitors on Vip3Aa-treated Sf9 cells. Sf9 cells were pretreated with the inhibitors, CA-074me (10 μM), Z-Phe-Tyr-CHO (10 μM), or pepstatin (15 μM) 2 h before Vip3Aa was added. (**A**) The apoptotic rate of Vip3Aa-treated cells incubated with or without inhibitors. The apoptotic rate was evaluated by Annexin V-FITC/PI stains. (**B**) The cell viability of Vip3Aa-treated cells incubated with or without inhibitors. The cell viability was measured by a CCK-8 assay. Significant tests from the corresponding controls (without Vip3Aa treatment) are indicated by NS, not significant, ** *p* < 0.01.

Meanwhile, we detected the effects of cathepsins (B, L, and D) on Vip3Aa-induced apoptosis and toxicity using the inhibitors CA-074me (a cathepsin B inhibitor), Z-Phe-Tyr-CHO (a cathepsin L inhibitor) and pepstatin (a cathepsin D inhibitor). As illustrated in Figure 6A, the percentage of late apoptotic cells was 0.1%, which rose to 12.56% after 48 h of Vip3Aa treatment. Z-Phe-Tyr-CHO and pepstatin reduced the percentage of late apoptotic cells from 12.56% to 1.7% and 1.44%, respectively. However, CA-074me, a cathepsin B inhibitor, had a little impact on the late apoptotic rate. Compared with Vip3Aa used alone, when Z-Phe-Tyr-CHO was used, there was a little effect on the proportion of early apoptotic cells, but the proportion of late apoptotic cells decreased significantly. However, when pepstatin was used, the proportion of early and late apoptotic cells all decreased significantly. These results suggested that cathepsin D contributes to Vip3Aa-induced apoptosis more than cathepsin L, and cathepsin D plays a more important role in apoptotic signal transduction and enhancement.

We also detected the effect of inhibitors on cell livability. The results showed that Z-Phe-Tyr-CHO and pepstatin increased cell livability from 51.3% to 75.1% and 77.8%, respectively (Figure 6B). However, CA-074me had little effect on cell viability. These results suggested that cathepsins (L and D) play a critical role in Vip3Aa-induced cell death rather than cathepsin B. 

### 2.6. Effects of Inhibition of Cathepsins (L and D) on Cytochrome c Distribution and Caspase-9 and -3 Activity

**Figure 7:**
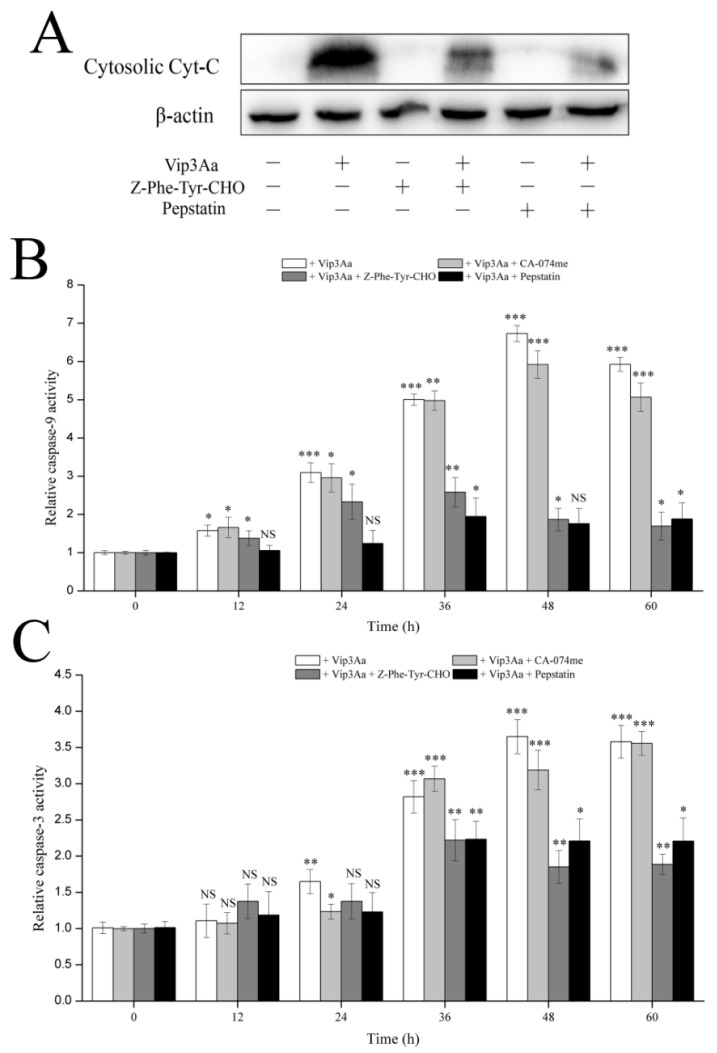
Effects of cathepsins inhibitors on caspases activity and cytochrome c release. (**A**) Impacts of cathepsin (L and D) inhibitors on cytochrome c distribution. The original pictures of Western blotting (with protein maker) are shown in Supplementary Materials (Figure S3). (**B**) Impacts of cathepsin inhibitors on caspase-9 activity. (**C**) Impacts of cathepsin inhibitors on caspase-3 activity. Significant tests from the corresponding controls (without Vip3Aa treatment) and densitometry of the protein bands are indicated by NS, not significant, * *p* < 0.05, ** *p* < 0.01, and *** *p* < 0.001.

Cytochrome c plays a vital role in apoptosis when the mitochondrial pathway is the executor. Thus, we investigated whether the cathepsin (L and D) inhibitors, Z-Phe-Tyr-CHO and pepstatin, could impact the release of cytochrome c. As shown in Figure 7A, the cathepsin (L and D) inhibitors, especially pepstatin, could reduce cytochrome c release. These results indicated that the function of lysosomes affected Vip3Aa-induced mitochondrial dysfunction.

Vip3Aa induced apoptosis in Sf9 cells in a caspase-dependent mode [15]. Mitochondrial membrane permeation and the release of cytochrome c from mitochondria activate caspase-9. Caspase-9 further activates caspase-3 and induces apoptosis. To further confirm the influence of lysosomes on Vip3Aa-induced mitochondrial pathway, we detected the caspase-9 and -3 activity without or with the cathepsin inhibitors. As shown in Figure 7B,C, the activity of caspase-9 and -3 increased from 24 h, peaked at 48 h after Vip3Aa treatment without cathepsin inhibitor. However, the caspase-9 and -3 activity decreased significantly after Vip3Aa treatment for 36 h with Z-Phe-Tyr-CHO or pepstatin. As expected, CA-074me had a little impact on caspase activity, especially on caspase-3. These findings indicated that lysosomes are involved in Vip3Aa-induced apoptosis and that cathepsins (L and D) have a vital impact on the Vip3Aa-induced mitochondrial pathway.

## 3. Discussion

Vip3Aa is a potent toxin against lepidopteran pests, especially to some pests of Noctuidae which are insensitive to ICPs. Recently, studies have shown that Vip3Aa could exert cytotoxicity by triggering apoptosis of insect cells and tissues besides formatting pores [3,5,14,15]. However, the specific mechanism of apoptosis induced by Vip3Aa remains unclear. Hence, we dissected the mechanism of mitochondrial pathway in Vip3Aa-induced apoptosis and found the lysosomes play a crucial role in Vip3Aa-induced apoptosis. The action mechanism of Vip3Aa found in Sf9 cells may also occur in insect intestinal epithelial cells.

Apoptosis includes two important pathways: the extrinsic pathway and the intrinsic pathway, mediated by the death receptor and mitochondria, respectively [24]. Jiang et al. [15] found that mitochondrial membrane potential decreased in Vip3Aa-treated Sf9 cells. We confirmed that Vip3Aa reduced Sf9 cell viability and caused mitochondria morphological alterations, which included swelling and disrupted cristae structure. In our study, we also found that Vip3Aa-induced apoptosis was mediated by mitochondrial dysfunction caused by loss of *ΔΨm*, which subsequently led to cytochrome c release and caspase-9 and -3 activation. Bcl-2 family proteins and caspases are involved in programmed cell death by regulating the protein levels. Moreover, the trends of capase-3 activity were consistent with those of caspase-9 in Vip3Aa-treated Sf9 cells. These results supported that the intrinsic mitochondrial pathway is involved in Vip3Aa-induced apoptosis in Sf9 cells.

Studies have indicated that the extrinsic pathway can be triggered by activating the death receptor on the cell membrane [25]. Additionally, the receptor-mediated pathway contains two types of mechanisms. In type I cells, the extrinsic apoptotic pathway leads to the activation of caspase-8, which directly activates effector caspases (caspase-3), causing apoptosis [26]. Nevertheless, in type II cells, the two apoptotic pathways, i.e., extrinsic pathway and intrinsic pathway, can be linked by caspase-8, which can cleave non-activated Bid protein into truncated Bid (tBid) [27]. tBid could activate Bax, resulting in cytochrome c release and caspase-3 activation [28]. To explore whether the death receptor pathway involves Vp3Aa-induced apoptosis, we also detected the activity of caspase-8 (Figure S4). The caspase-8 activity increased a little bit from 12 to 36 h, but it was lower than that of untreated cells from 48 h. This suggested that caspase-8 might not contribute to the activation of caspase-3. Moreover, the two Vip3Aa receptors SR-C and FGFR did not contain the death domain [9,10]. Jiang et al. [9] revealed the toxicity of Vip3Aa to Sf9 cell correlates with its endocytosis mediated by Sf-SR-C and the internalization is essential for Vip3Aa to exert its toxic effects. On this basis, we further showed that internalized Vip3Aa impacted the features of lysosomes. These results suggested that Vip3Aa-induced apoptosis might involve the internalization of Vip3Aa and the denaturation of lysosomes rather than the death receptor-mediated pathway.

In this study, we found that the abundant colocalization of Vip3Aa and lysosome in Sf9 cells and Vip3Aa had a distinct effect on lysosome morphological and physicochemical properties. Thus, we thought the lysosomes contain the Vip3Aa, and the deformed lysosomes might be the consequence of Vip3Aa action. Duve et al. [29] put forward that lysosomes play a role in apoptosis in 1966. A new death theory, the lysosome–mitochondria axis, mainly emphasized that hydrolytic enzymes were released to the cytosol from the lysosome when lysosomal membranes were permeabilized, resulting in mitochondrial dysfunction, cytochrome c release, and caspase activation. Many studies had reported that cathepsins could be involved in the signaling of apoptosis. Cathepsin D can activate Bax and the active form of Bax translocates to the mitochondria, leading to the opening of transition pores on the mitochondrial membrane, which cause apoptosis factors such as cytochrome c to be released [16]. Another study indicated that cathepsin L acts as a death signal integrator and cytosolic cathepsin L regulated the cytochrome c release and caspase-3 activity in cervical cancer cells [30]. In this study, the results (Figure 6 and Figure 7) showed that cathepsin (L and D) inhibitors could protect Sf9 cells from Vip3Aa and suppress cytochrome c release and inhibit the caspase-9 and -3 activity, suggesting that cathepsins (L and D) played a significant role in Vip3Aa-induced cell death. Moreover, some studies found that cathepsin B associated with programmed cell death of the fat body cells in the process of silkworm metamorphosis [31]. However, there was a little effect of cathepsin B on the Vip3Aa-treated Sf9 cells. As for the role of cathepsins (L and D), they may be released to the cytoplasm and could cleave Bid to tBid, and the latter triggers the mitochondrial outer membrane permeabilization, resulting in mitochondria dysfunction. On the other hand, cathepsins (L and D) may contribute to activating Vip3Aa in lysosomes. Many studies indicated that cathepsins were involved in the physiological reaction of insects, but the exact mechanism is unclear. In this study, lysosomes were firstly found to be involved in the process of Vip3Aa-induced apoptosis. However, the mechanism needs further investigation. 

We found the caspase inhibitor (Z-VAD-FMK) could not protect all the Sf9 cells from Vip3Aa (Figure S5). This result suggested that some other apoptosis-independent cell death mechanisms, such as pore-forming, might be involved in cell death caused by Vip3Aa [3,8]. Some microbial toxins, such as aerolysin produced by *Aeromonas hydrophila* and α-toxin generated by *Staphylococcus aureus*, could contribute to pore-forming and apoptosis in their target cells [32]. Similarly, Vip3Aa may cause insect cell death through two mechanisms at the same time. 

In conclusion, in Sf9 cells, we showed that the mitochondria pathway serves as the executor in Vip3Aa-induced apoptosis, while lysosomes are involved in Vip3Aa-induced mitochondrial dysfunction and apoptosis. Our findings can provide a venue for promoting the knowledge of Vip3Aa action.

## 4. Materials and Methods 

### 4.1. Cell Culture and Reagents

The Sf9 cells were cultured in Sf-900 II SFM medium (Gibco, 10902088, Grand Island, NY, USA) supplemented with 6% FBS (GIBCO, Grand Island, NY, USA), at 28 ℃. RIPA buffer (#9806S), and antibodies against Bax (#2772), Bcl-2 (#15071), caspase-9 (#9508), cytochrome c (#11940), and β-actin (#8457) were obtained from Cell Signaling Technology (Beverly, MA, USA). Acridine orange (#A8120) was purchased from Solarbio Life Science (Beijing, China). Antibody against Bcl-XL (#abs131907) was purchased from Absin Bioscience (Shanghai, China). Antibody against Caspase-3 (#A2156) was purchased from ABclonal (Wuhan, China). Goat anti-mouse IgG-HRP conjugate (#sc-2005) or anti-rabbit (#sc-2357) was purchased from Santa Cruz (Santa Cruz, TX, USA). DCFH-DA (#S0033) and Rhodamine123 (#C2007) were purchased from Beyotime Biotechnology (Nanjing, China). LysoSensor™ Green DND-189 (#40767ES50) was purchased from Yeasen Biotechnology (Shanghai, China).

### 4.2. Vip3Aa Purification

pET-28a (+) vector was used to construct a recombinant expression plasmid. The BL21 (DE3) strains transferred with pET28a-Vip3Aa were cultured to OD600 0.8–1.0, and IPTG (0.5 mM) were used to induce the protein expression at 16 °C for 12–16 h. Then, the cells were collected, broken by ultrasonication, and purified using Ni Sepharose^TM^ affinity column. The Vip3Aa was dialyzed in a buffer containing 25 mM Tris-Hcl (pH 7.4) and 150 mM NaCl at 4 °C. The result of purified Vip3Aa was shown in Figure S6. The concentration of Vip3Aa was measured via the protein-dye method of Bradford. BSA was used as a standard protein. The full-length Vip3Aa was used directly in Sf9 cells.

### 4.3. Cell Viability Assay

The cell viability was detected using the CCK-8 Counting Kit (Dojindo, Kumamoto, Japan). Cell suspensions (100 μL, 2.5 × 10^5^ cells/mL) were pipetted into a 96-well plate and incubated overnight at 28 ℃. Then, Vip3Aa was added into the suspensions. The cells were exposed to Vip3Aa for 24, 48, 60, and 72 h. The final concentration of Vip3Aa was 10, 20, 30, 40, and 50 μg/mL. Sf-900 II SFM medium and cell suspensions without Vip3Aa were used as blank group and control group, respectively. Then, CCK-8 reagent (10 μL) was added and incubated in darkness for 2–4 h at 28 ℃. The results were monitored at 450 nm using a microplate reader (PerkinElmer, Boston, MA, USA). The experiments were performed six times. Cell viability was the ratio of absorbance of Vip3Aa-treated group/control group.

### 4.4. Vip3Aa Subcellular Localization in Sf9 Cells

The cells were exposed to Vip3Aa-RFP for 0, 2, 4, and 6 h. Then, the cells were incubated with Sf-900 II SFM medium containing 1 μM LysoSensor™ Green DND-189 at 28 ℃ in the darkness for 45 min. The cells were washed with phosphate-buffered saline (PBS) (pH 7.4) three times and imaged with a Zeiss LSM710 fluorescence microscope.

### 4.5. Transmission Electron Microscopy (TEM)

Cell suspensions (5 × 10^5^–1 × 10^6^ cells/mL) were incubated overnight in 25 cm^2^ flasks. The cultures were exposed to Vip3Aa (final concentration, 40 μg/mL) for 12, 24, 36, and 48 h, respectively. Transmission electron microscope (TEM) was used to observe and record the ultrastructure of Sf9 cells. The cells were fixed in 2% paraformaldehyde and 2.5% glutaraldehyde in phosphate-buffered saline (PBS, pH 7.4) for 2 h after washing with PBS three times. Then, the fixed cells were treated with 1% osmic acid (OsO_4_) at 25 ℃ for 1 h after washing with PBS. The cell samples were dehydrated in different concentration ethanol solutions, soaked, and embedded in EPON812. Ultrathin (60 nm) sections were cut and counterstained with lead citrate and uranyl acetate. The sections were observed with TEM (JEOL-1200EX).

### 4.6. Measurement of Intracellular ROS and Mitochondrial Membrane Potential (ΔΨm) 

DCFH-DA and Rhodamine 123 were utilized to measure intracellular ROS [33] and *ΔΨm*, respectively. The cells were exposed to Vip3Aa (final concentration, 40 μg/mL) for different times. Then, the cells were incubated with Sf-900 II SFM medium containing 10 mM of DCFH-DA or 50 nM Rhodamine 123 at 28 ℃ in the darkness for 30 min. The cells were washed with PBS (pH 7.4) three times and imaged with a Zeiss LSM710 fluorescence microscope.

### 4.7. Total Protein and Cytosolic Protein Extraction

The cells were exposed to Vip3Aa (final concentration, 40 μg/mL) for different times. The cells were lysed in 350 μL RIPA buffer with 1 mM PMSF and incubated on ice for 15 min after washing with PBS (pH 7.4) three times. The suspension was centrifuged at 12,000× *g* for 15 min. Then, the supernatant, which was the total protein extraction, was collected carefully.

Cells were washed and collected by centrifugation at 200× *g* for 5 min. The cells were resuspended with 500 μL isotonic buffer (IB, 10 mM HEPES, 200 mM mannitol, 1 mM EGTA, 70 mM sucrose). The cell suspension was centrifuged at 3000× *g* for 5 min. The collected cells were resuspended in 500 μL IB with 20 mM NaF, 20 mM Na_3_VO_4_ and 1 mM PMSF. Then, 26-G needles were used to homogenize the cell suspension, which was passed through 14 times and stood on ice for 5 min. The suspension was centrifuged at 4 ℃, 10,000× *g* for 15 min. Centrifugation sediment contains lysosomes and mitochondria. Then, the supernatant was diverted to a fresh cold centrifuge tube and centrifuged at 4 ℃, 14,000× *g* for 30 min in an ultracentrifuge. The supernatant, which was the cytosolic protein extraction, was collected carefully.

### 4.8. Quantitative Real-Time PCR 

Trizol reagent (Invitrogen, Carlsbad, CA, USA) was used to extract total RNA. Chloroform and isopropanol were used to isolate RNA. The primers used in this study are listed in Table 1. A Primescript^TM^ RT reagent kit with gDNA Eraser (TakaRa, Dalian, China) was utilized to reverse-transcribe RNA. The quantitative real-time PCR was performed with SYBR^®^ Premix Ex Taq™ (TakaRa, Dalian, China) in an ABI Prism 7900HT Real-Time PCR System (Applied Biosystems, Carlsbad, CA, USA). *GAPDH* was used as the control for normalization by the 2^-ΔΔCt^ method [34]. 

### 4.9. Western Blotting Analysis

A BCA Protein Assay kit (TIANGEN, Beijing, China) was used to test the concentrations of protein samples. Next, 12% SDS-PAGE electrophoresis was utilized to separate the target proteins, which were transferred onto the PVDF membrane. Primary antibodies were anti-caspase-3 (1:1000), anti-caspase-9 (1:500), anti-Bcl-XL (1:500), anti-Bcl-2 (1:500), anti-Bax (1:500), anti-cytochrome c (1:500), anti-cathepsin L (1:500), and anti-β-actin (1:500). Mouse anti-rabbit IgG-HRP (1:1000) and goat anti-mouse IgG-HRP (1:1000) were the secondary antibodies. Finally, the PVDF membranes were visualized using Immobilon Western Chemiluminescent HRP Substrate (Millipore, Milan, Italy).

### 4.10. Acridine Orange (AO) Staining Analysis

The AO staining analysis was performed by flow cytometry (Becton Dickinson, USA). The Sf9 cells were treated with Vip3Aa (final concentration, 40 μg/mL) for different times. Then, the cells were incubated with Sf-900 II SFM medium containing 5 μg/mL AO at 28 ℃ in the darkness for 10 min. The stained cells were used to analyze the fluorescence distribution (FL1-H/FL3-H) after washing with PBS (pH 7.4) three times. After adjusting the fluorescence compensation of the channels, the number of recorded cells was 10,000.

### 4.11. Lysosomal pH Assay

The LysoSensor Yellow/Blue DND-160 (Life Technologies, Carlsbad, CA), a lysosomal pH indicator, was used to measure the Sf9 cells lysosomal pH. Cell suspensions (100 μL, 2.5 × 10^5^ cells/mL) were pipetted into a 96-well black plate. All the cells were incubated with Sf-900 II SFM medium containing 5 μM fluorescent probe at 28 ℃ in the darkness for 5 min. Then, the cells were washed and cultured in an MES calibration buffer (1.2 mM MgSO_4_, 115 mM KCl, 5 mM NaCl, 25 mM MES, pH 3.5–6.0) containing 10 μM monensin and 10 μM nigericin. The fluorescence value (Ex340 nm/Em540 nm and Ex380 nm/Em540 nm) was monitored by a microplate reader (PerkinElmer, Boston, MA, USA). The pH calibration curve was generated using ratios of the two light emission intensities and the corresponding pH value. To find the effect of Vip3Aa on lysosomal pH, the Vip3Aa-treated cells were incubated with Sf-900 II SFM medium containing 5 μM fluorescent probe at 28 ℃ in the darkness for 5 min, washed, and resuspended in MES buffer (pH 7.0) and detected using a microplate reader (PerkinElmer, Boston, MA, USA). The lysosomal pH was estimated using the ratios and the pH calibration curve. Sf-900 II SFM medium and cell suspensions without Vip3Aa were used as blank group and control group, respectively.

### 4.12. Apoptosis Assay

Sf9 cells were treated with Vip3Aa (final concentration, 40 μg/mL) for 48 h with/without cathepsins inhibitor for 2 h. We evaluated the proportion of apoptotic cells using the FITC annexin V apoptosis detection kit I (BD Biosciences, USA). Cells without Vip3Aa-treated were used as a control group. After washing twice with PBS (100 × g, 5 min), cells incubated with 1 × binding buffer containing FITC annexin V at 28 ℃ in the darkness for 30 min. Then, 1 × binding buffer containing propidium iodide was added to each sample. After incubating at 28 ℃ in the darkness for 5 min, the cells were monitored with a flow cytometer (Becton Dickinson, Franklin Lakes, NJ, USA).

### 4.13. Caspase Activity Analysis

Sf9 cells were treated with Vip3Aa (final concentration, 40 μg/mL) for different time with/without cathepsins inhibitor for 2 h. Caspase-Glo^®^ assay kit (Promega, Madison, WI, USA) was utilized to determine caspase activity. Cell suspensions (100 μL, 2.5 × 10^5^ cells/mL) were pipetted into a 96-well white plate and incubated overnight at 28 ℃. Then, Vip3Aa was added into the suspensions. The cells were exposed to Vip3Aa for 12, 24, 36, 48, 60, and 72 h. Sf-900 II SFM medium and cell suspensions without Vip3Aa were used as blank group and control group, respectively. Caspase-Glo^®^ Reagent was prepared according to the protocol and all the operations should be performed in the darkness. Equilibrate the reagent and plates to room temperature. Caspase-Glo^®^ Reagent (100 µL) was added to the plates containing cells in Sf-900 II SFM medium. A plate shaker was used to mix the plates containing cells and reagent at 300–500 rpm for 0.5–2 min. Then, the plates were incubated at room temperature for 2 h. Finally, the luminescence of each plate was detected using a microplate reader (PerkinElmer, Boston, MA, USA).

### 4.14. Statistical Analysis

The results were obtained from at least three independent experiments. The densitometry values were evaluated by the software Image J. Origin 8.0 (OriginLab, Northampton, MA, USA) was used to draw the graphs. The significance was tested by one-way analysis of variance utilizing Student t test. If *p*-value ≤0.05, the results were considered significant.

## Figures and Tables

**Table 1:** Primers used in this study.

Primers	Primer Sequence
qCathepsin B-F	5′-GAAGTGAGGGACCAAGGAT-3′
qCathepsin B-R	5′-TCTGCGGAGAAGTGGAAAT-3′
qCathepsin L-F	5′-CAGGGTGATGAGGAGAAGC-3′
qCathepsin L-R	5′-TCGGTGGACGAGCAGTT-3′
qCathepsin D-F	5′-CAGGGGCTGGTGAAGCCA-3′
qCathepsin D-R	5′-CACGTACGTGAAGTTGCC-3′
qGAPDH-F	5′-GTGCCCAGCAGAACATCAT-3′
qGAPDH-R	5′-GGAACACGGAAAGCCATAC-3′

## Supplementary Materials

The following are available online at https://www.mdpi.com/2072-6651/12/2/116/s1, Figure S1: Impacts of Vip3Aa in Sf9 cells on the cytochrome c distribution and the level of mitochondria-associated proteins. (A) Cytochrome c distribution in mitochondria and cytosol was detected by Western blotting. (B) Cytochrome c distribution in Sf9 cells after various treatments. CsA (5 μM) or BKA (10 μM) was pretreated the Sf9 cells for 2 h before adding Vip3Aa. (C) Mitochondria-associated protein levels in Sf9 cells were tested using Western blotting. (D, E, and F) Densitometry analysis of (A) and (C). Figure S2: Effect of Vip3Aa on cathepsins mRNA level in Sf9 cells at different time. Figure S3: Effects of cathepsin (L and D) inhibitors on cytochrome c distribution. (A) cytochrome c distribution in cytosol was detected by Western blotting. (B) Densitometry analysis of (A). Figure S4: Effects of Vip3Aa on caspase-8 activity. Significant tests from the corresponding controls (without Vip3Aa treatment) are indicated by NS, not significant, **p* < 0.05, ***p* < 0.01, ****p* < 0.001. Figure S5: Effects of caspase-3 inhibitor on Sf9 cell viability. Significant tests from the corresponding controls (without Vip3Aa treatment) are indicated by **p* < 0.05, ***p* < 0.01, ****p* < 0.001. Figure S6: Result of purified Vip3Aa detected by SDS-PAGE electrophoresis analysis. M, protein maker 26619.
