# Supplementary material for: Mitochondria and Lysosomes Participate in Vip3Aa-Induced Spodoptera frugiperda Sf9 Cell Apoptosis
Source: Toxins (Basel). 2020 Feb 13;12(2):116. doi: 10.3390/toxins12020116 (PMC7076775; doi:10.3390/toxins12020116)
Supplement: Supplementary file 1 [file toxins-12-00116-s001.pdf]

Supplementary Materials: Mitochondria and Lysosomes Participate in Vip3Aa-Induced Spodoptera frugiperda Sf9 Cell Apoptosis

Xiaoyue Hou, Lu Han, Baoju An, Yanli Zhang, Zhanglei Cao, Yunda Zhan, Xia Cai, Bing Yan and Jun Cai

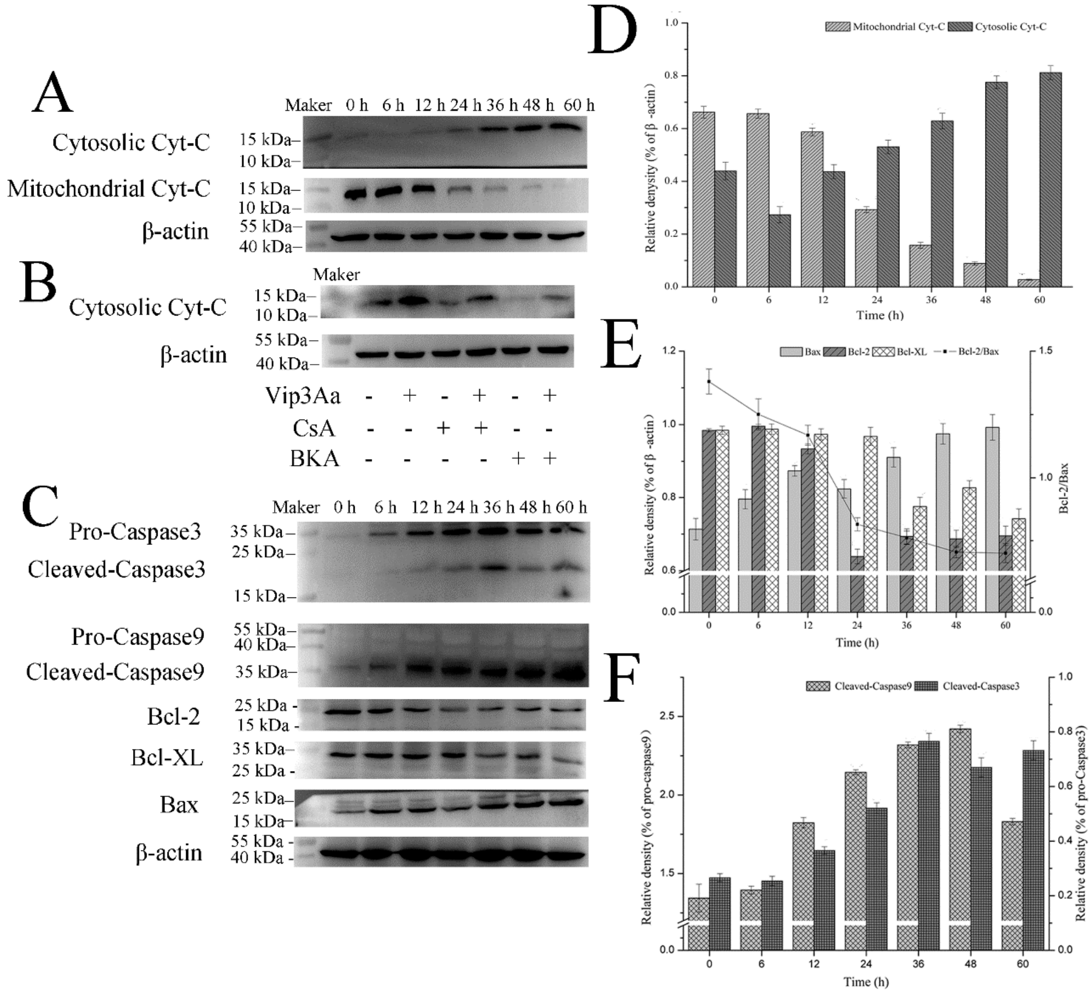

Figure S1. Impacts of Vip3Aa in Sf9 cells on the cytochrome c distribution and the level of mitochondria-associated proteins. (**A**) Cytochrome c distribution in mitochondria and cytosol was detected by Western blotting. (**B**) Cytochrome c distribution in Sf9 cells after various treatments. CsA (5 μM) or BKA (10 μM) was pretreated the Sf9 cells for 2 h before adding Vip3Aa. (**C**) Mitochondria-associated protein levels in Sf9 cells were tested using Western blotting. **(D, E**, and **F**) Densitometry analysis of (**A**) and (**C**).

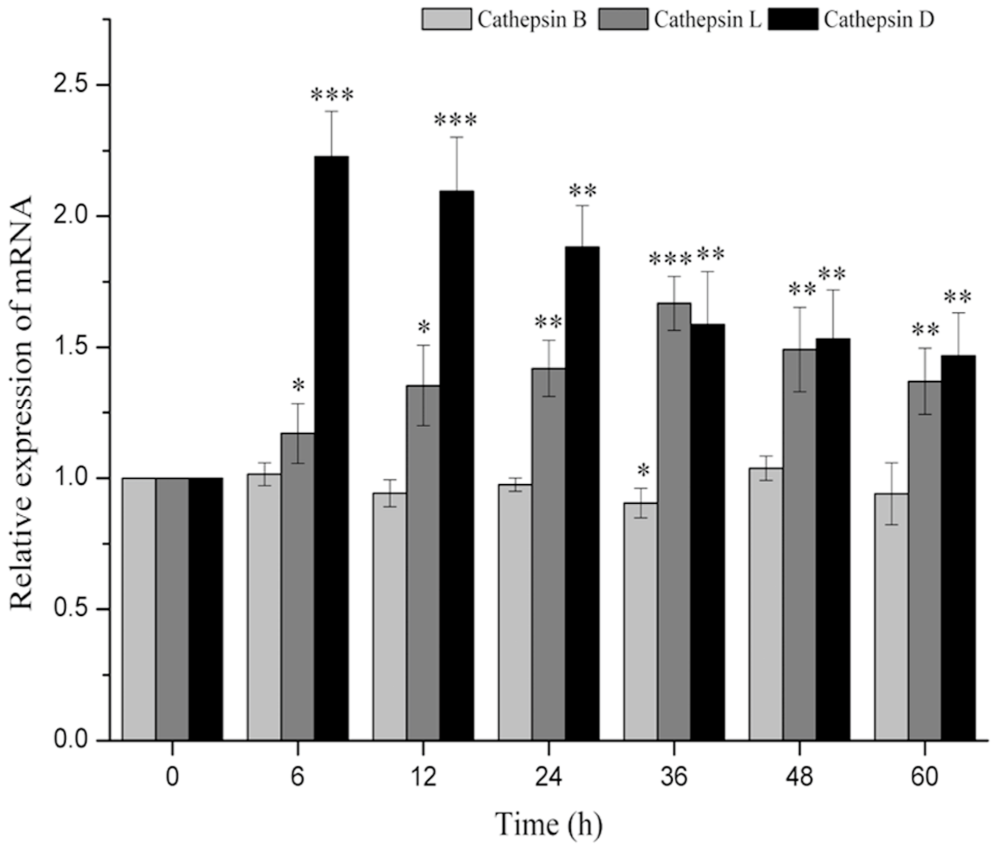

Figure S2. Effect of Vip3Aa on cathepsins mRNA level in Sf9 cells at different time.

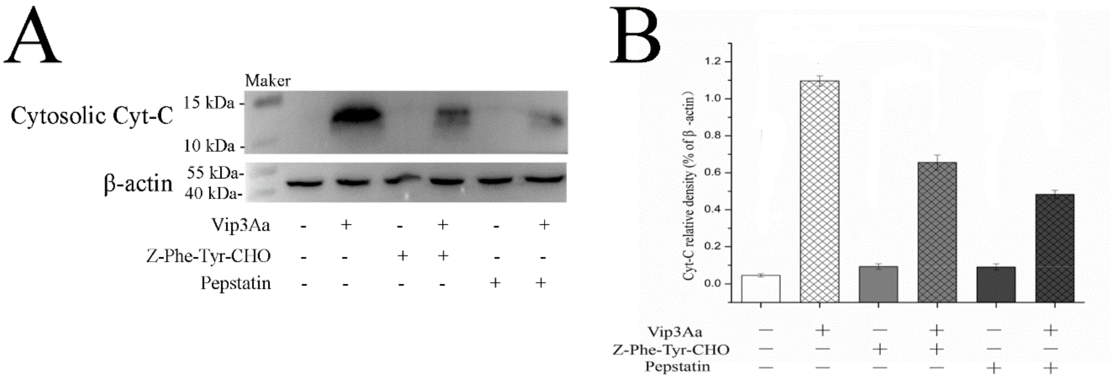

Figure S3. Effects of cathepsin (L and D) inhibitors on cytochrome c distribution. (**A**) cytochrome c distribution in cytosol was detected by Western blotting. (**B**) Densitometry analysis of (**A**).

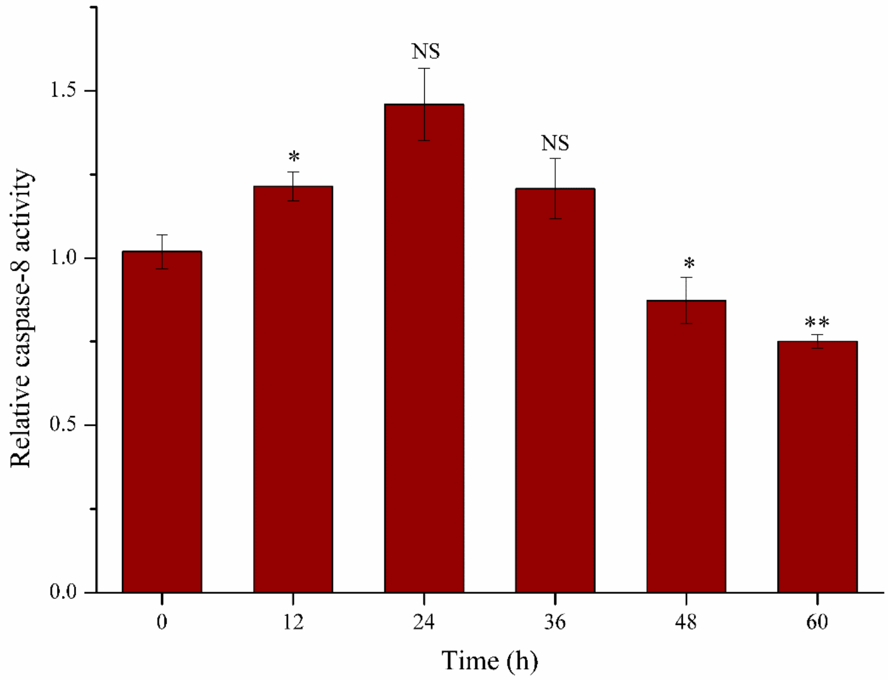

Figure S4. Effects of Vip3Aa on caspase-8 activity. Significant tests from the corresponding controls (without Vip3Aa treatment) are indicated by NS, not significant, *$p < 0.05$, **$p < 0.01$, ***$p < 0.001$.

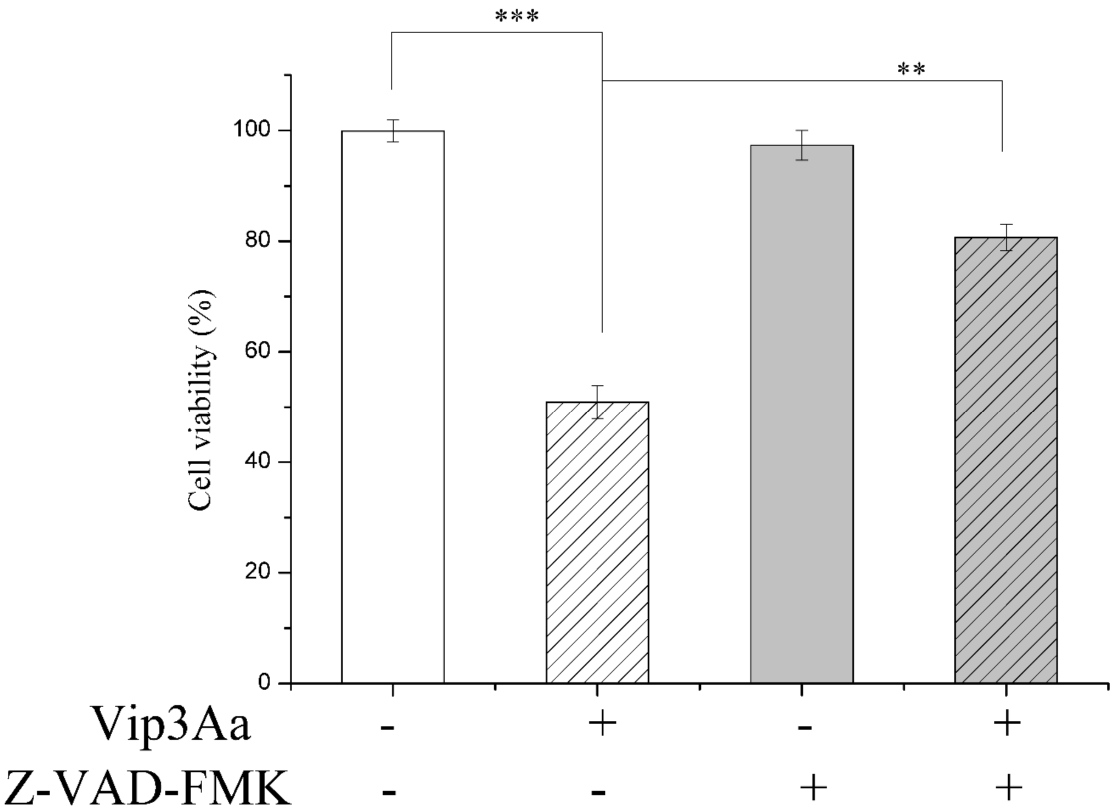

Figure S5. Effects of caspase-3 inhibitor on Sf9 cell viability. Significant tests from the corresponding controls (without Vip3Aa treatment) are indicated by *$p < 0.05$, **$p < 0.01$, ***$p < 0.001$.

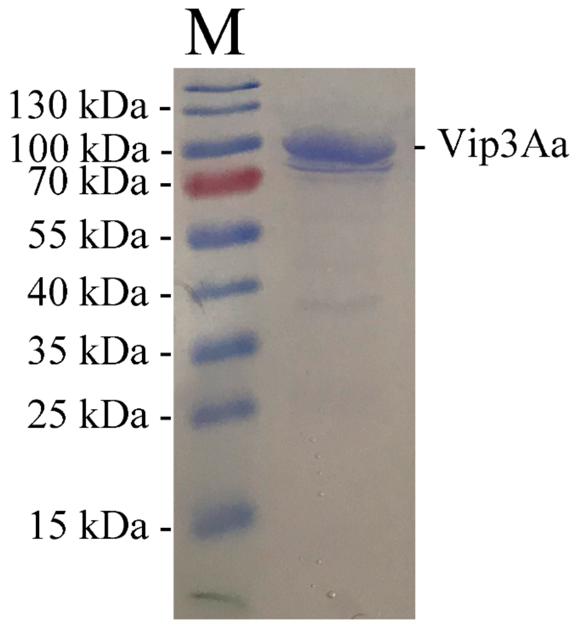

Figure S6. Result of purified Vip3Aa detected by SDS-PAGE electrophoresis analysis. M, protein maker 26619.
